# DO-PI-EATCNet: Efficient-Attention- and Dream-Optimization-Based Channel Selection for EEG Motor Imagery Classification

**DOI:** 10.3390/s26113336

**Published:** 2026-05-24

**Authors:** Xiaoyan Shen, Hongkui Zhong, Yujie Gu, Ruiqing Han

**Affiliations:** 1 School of Information Science and Technology, Nantong University, Nantong 226019, China; 2 School of Civil Engineering, Central South University, Changsha 410083, China

**Keywords:** MI-EEG classification, fractional-order difference temporal consistency loss, multi-population dream optimization algorithm, temporal channel cascaded collaborative attention, latent-projected attention

## Abstract

Existing deep-learning-based motor imagery (MI) electroencephalogram (EEG) decoding methods face challenges in generalizing across sessions and providing channel-level physiological interpretability. These limitations hinder the practical application of MI-EEG systems. Accordingly, DO-PI-EATCNet (Dream-Optimization-Enhanced, Physics-Inspired, Efficient-Attention Temporal Channel Network) is proposed to improve generalization and interpretability in MI-EEG classification. Unlike models that simply combine multiple components, DO-PI-EATCNet assigns distinct roles to feature representation, temporal channel modeling, temporal regularization, and channel compactness. Latent-Projected Attention (LPA) enhances spatiotemporal discriminability by aligning attention in a low-dimensional latent space, and Temporal Channel Cascaded Collaborative Attention (TCCA) refines dependencies between time and channels. Fractional-Order Difference Temporal Consistency Loss (FD-TCL) is introduced as a neurodynamics-inspired temporal regularizer to reduce high-frequency fluctuations in prediction sequences and improve within-subject cross-session prediction stability. The Multi-Population Dream Optimization Algorithm (MPDOA) is used for channel selection to obtain a compact EEG channel subset and reduce computational load, although it introduces a slight accuracy decrease compared with the uncompressed full model. Under a within-subject cross-session protocol on the BCI Competition IV-2a four-class MI dataset, the final compact model achieves an average accuracy of 84.4% and Cohen’s *κ* of 0.790, outperforming the reimplemented baselines. Compared with the uncompressed LPA-TCCA-FD-TCL variant, MPDOA slightly decreases accuracy from 84.9% to 84.4%, but reduces EEG channels from 22 to about 15 and decreases MACs by 27%. Scalp topographies and selected-channel visualizations provide qualitative support for channel-level anatomical plausibility, as the selected electrodes are mainly located over expected sensorimotor-related regions, while t-SNE offers a descriptive visualization of the learned feature distributions.

## 1. Introduction

With the rapid development of brain–computer interface (BCI) technology, motor imagery (MI) classification based on electroencephalogram (EEG) signals has shown broad application prospects in rehabilitation medicine, human–computer interaction, and neural engineering [1,2]. MI is a noninvasive interaction modality in which external devices are controlled solely by brain intention without actual limb movements [3]. This technology provides a new means of interaction for individuals with severe disabilities, people with limb impairments, and complex systems that require intelligent control, and it has important clinical and industrial value [4]. Due to its high temporal resolution, convenient acquisition, and physiological safety, EEG has become a commonly used signal source in BCI systems.

However, MI-EEG decoding remains challenging in practical applications. Intrinsic properties of EEG signals, such as nonstationarity, low signal-to-noise ratio, and pronounced inter-subject variability, make signal decoding and classification difficult [5,6]. In motor imagery tasks, task-related neural responses are usually weak and are easily affected by background noise and individual differences. Therefore, stable feature extraction and reliable discrimination remain key difficulties that restrict further improvement of MI-EEG classification performance, especially in multiclass and cross-session scenarios [7,8].

Although traditional machine learning methods and deep-learning-based models have improved MI-EEG classification performance, several limitations remain. Many existing methods still rely on full-channel EEG inputs, which may introduce redundant information and increase computational burden. In addition, end-to-end deep learning models often provide limited channel-level physiological interpretability and may be sensitive to cross-session distribution shifts. Therefore, it is necessary to develop a model that can jointly improve spatiotemporal discriminative representation, temporal stability, channel compactness, and computational efficiency.

To address these issues, a classification model for complex multiclass motor imagery tasks is proposed, denoted as DO-PI-EATCNet, which refers to Dream-Optimization-Enhanced, Physics-Inspired, Efficient-Attention Temporal Channel Network. The model integrates key innovations at different stages of the processing pipeline, including feature representation, temporal channel modeling, temporal regularization, and channel selection. The backbone of DO-PI-EATCNet is an attention-enhanced temporal convolutional network (ATCNet), which is complemented by four modules: Latent-Projected Attention (LPA), Temporal Channel Cascaded Collaborative Attention (TCCA), Fractional-Order Difference Temporal Consistency Loss (FD-TCL), and the Multi-Population Dream Optimization Algorithm (MPDOA).

Instead of assuming that all components contribute to the same performance aspect, DO-PI-EATCNet assigns distinct functional roles to different modules. LPA and TCCA are mainly designed to improve discriminative spatiotemporal representation. FD-TCL is introduced as a neurodynamics-inspired temporal regularization term to reduce high-frequency prediction fluctuations and enhance cross-session stability. MPDOA is primarily used to obtain a compact EEG channel subset and reduce input-side computational load. Therefore, the final model should be interpreted as a trade-off among classification accuracy, temporal stability, channel reduction, and computational efficiency. The main contributions of this work can be summarized as follows:

(1) At the representation and structural levels, LPA and TCCA are integrated into the ATCNet backbone to improve spatiotemporal discriminative representation. LPA performs attention alignment in a low-dimensional latent subspace, while TCCA models temporal channel dependencies in a cascaded manner. These two modules mainly contribute to the improvement of classification accuracy in complex multiclass MI-EEG decoding.
(2) At the channel level, an MPDOA-based automatic channel optimization strategy is proposed. This strategy searches for a compact subject-specific channel subset and reduces the number of input EEG channels from 22 to about 15. MPDOA is positioned as an efficiency-oriented channel selection component, whose primary role is to improve channel compactness and reduce arithmetic computational load while preserving most of the classification performance.
(3) At the objective level, FD-TCL is designed as a neurodynamics-inspired fractional-order temporal regularizer. It provides a soft temporal consistency constraint that suppresses high-frequency fluctuations in prediction sequences and promotes smoother confidence evolution, thereby improving within-subject cross-session stability.
(4) At the experimental validation level, systematic experiments are conducted on the public BCI Competition IV-2a four-class MI dataset under a within-subject cross-session protocol. The proposed method is evaluated using accuracy, Cohen’s κ, confusion matrix, ablation studies, computational-complexity analysis, and visualization analyses. The results show that DO-PI-EATCNet achieves favorable classification performance through LPA, TCCA, and FD-TCL, while MPDOA provides a compact channel configuration with a slight accuracy decrease. Scalp topographies and selected-channel distributions are further used to assess channel-level anatomical plausibility.

The remainder of this paper is organized as follows. Section 2 reviews related work on existing MI-EEG classification methods, deep-learning-based EEG decoding, and EEG channel selection. Section 3 describes the overall framework of DO-PI-EATCNet and its key modules, including LPA, TCCA, FD-TCL, and MPDOA. Section 4 introduces the BCI Competition IV-2a four-class MI dataset, the data preprocessing pipeline, and the evaluation metrics. Section 5 presents the experimental settings and results, including comparisons with baseline methods, ablation studies, analysis of the impact of channel selection on computational cost, and visualization analysis. Section 6 concludes this study.

## 2. Related Work

In EEG signal classification tasks, feature selection and classifier design are generally considered two key components that determine classification performance. Traditional feature extraction methods mainly include time-domain features, such as mean and variance; frequency-domain features, such as power of *μ* and *β* bands; and time-frequency features, such as wavelet transform-based representations [9,10]. In addition, spatial filtering techniques such as common spatial patterns (CSPs) and filter bank CSP (FBCSP) construct discriminative spatial projections and can effectively enhance class separability [11,12]. Riemannian methods start with the geometric structure of covariance matrices and exhibit advantages in terms of robustness to noise and stability [13]. After feature construction is completed, traditional studies usually adopt machine learning classifiers such as support vector machine (SVM), linear discriminant analysis (LDA), and random forest to discriminate MI-EEG signals [14,15,16]. These methods involve relatively few parameters and provide high training efficiency. However, they usually follow a two-stage design of “feature extraction plus classifier”, which relies heavily on manual experience and makes it difficult to jointly model high-dimensional and nonlinear spatiotemporal patterns. In complex multiclass tasks and cross-session scenarios, their performance improvement gradually encounters a bottleneck.

In recent years, deep-learning-based classification models have achieved outstanding performance in MI-EEG classification [17]. Compared with traditional machine learning methods, deep networks can automatically learn discriminative feature representations and decision boundaries within a single model, and they are particularly advantageous in handling complex temporal, spatial, and spectral features. In convolutional and attention-based deep networks, Ai et al. [18] proposed a holographic convolutional attention network (HCANet), in which multi-scale convolutions and attention mechanisms are used to model temporal, frequency, and spatial features simultaneously. Zhao et al. [19] constructed a multi-branch deep temporal fusion network, in which attention mechanisms enhance the focus of the model on different feature channels. To further balance interpretability and discriminative power, Han et al. [20] designed a dual-prototype network in spatial-spectral and temporal domains (SST-DPN), where distance constraints are used to learn class centers and enhance robustness. Zhu et al. [21] employed spiking neural networks (SNNs) to construct an adaptive spiking convolutional network (ASCN), which provides energy efficiency while maintaining relatively high accuracy. Yang et al. [22] proposed an efficient domain-adaptive attention neural network (EDANet), in which domain alignment is combined with attention mechanisms to realize transfer learning across subjects. Wang et al. [23] built a multi-task gated parallel feature fusion network for multimodal deep feature modeling and effectively enhanced feature representation capacity.

Transformer-based models and their variants have also been introduced into MI-EEG decoding. Hamidi et al. [24] integrated a Transformer with a graph convolutional network (GCN) and proposed a Transformer-GCN model that can simultaneously capture temporal dependencies and topological structures of brain regions, thereby improving discriminability in motor imagery tasks. Fu et al. [25] proposed a hybrid architecture that combines convolution and Transformer, and a domain adaptation module was introduced so that stronger generalization ability can be obtained in cross-session scenarios. Overall, although deep learning has led to significant progress in MI-EEG classification, many existing methods rely mainly on end-to-end architectures and ignore explicit modeling of the physiological mechanisms of EEG signals. As a result, it is difficult to explain the decision bases of the model at the levels of channels, brain regions, and frequency bands. At the same time, these models tend to be sensitive to sample distribution shifts and may show considerable performance fluctuations, especially in cross-session tasks.

Apart from the network structure, channel-level optimization also has an important impact on MI-EEG classification performance. Various brain regions can exhibit different response strengths to specific motor imagery tasks, whereas conventional EEG recordings usually adopt multichannel full-scalp montages. This may lead to an accumulation of redundant information, increase computational burden, and mask key discriminative features [26]. Existing studies have shown that focusing on channels over sensorimotor areas can effectively enhance classification performance. By selecting an electrode subset that is closely related to the task, the input dimensionality can be compressed while signal quality and recognition accuracy are maintained or improved, so that a joint optimization of performance and efficiency can be achieved [27].

Several channel selection methods have been proposed for EEG-based MI classification. Faye et al. [28] constructed a graph-based EEG channel selection method (G-EEGCS) based on a graph neural network and evaluated channel importance by modeling topological relationships among brain regions. Sun et al. [29] proposed an end-to-end model based on a graph convolutional network and realized joint optimization of channel selection and classification during training by means of a channel attention mechanism. Zhang et al. [30] introduced a sparsity regularization strategy into a convolutional neural network architecture and proposed a motor imagery recognition method that combines automatic channel selection with deep learning. These studies provide useful ways to identify discriminative channels and improve the interpretability of MI-EEG models.

However, channel selection is still handled in a relatively implicit way in many current deep learning models. Full-channel input strategies are still widely adopted, and explicit modeling and controllable optimization of channel discriminability are often insufficient. Although some methods attempt to model channel weights by using attention mechanisms or sparsity constraints, these strategies are usually restricted by fixed network structures and cannot easily achieve adaptive and globally optimized channel selection. Therefore, the construction of a deep learning framework that provides automatic channel optimization while balancing classification performance, physiological plausibility, and computational efficiency remains a key problem in current MI-EEG recognition.

## 3. DO-PI-EATCNet Model

**Figure 1:**
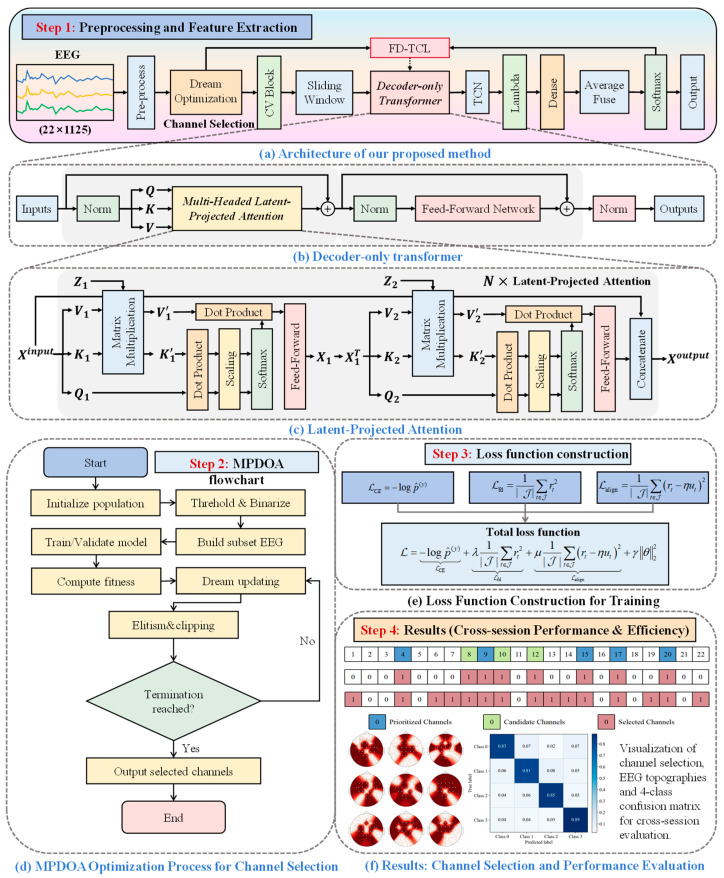
Overall architecture of the DO-PI-EATCNet model.

To improve within-subject cross-session decoding accuracy while reducing the number of channels and supporting channel-level anatomical plausibility, a lightweight framework named DO-PI-EATCNet is constructed in this section. As illustrated in Figure 1a, raw EEG signals are first processed by a preprocessing module (Pre-process). An MPDOA module then performs channel selection, and a convolutional feature extraction block (CV Block) extracts initial joint temporal-frequency features. Under a sliding window scheme (Sliding Window), these representations are partitioned into local segments and are fed into a decoder-only Transformer for deep modeling. Within this Transformer, the core computation unit consists of an LPA and a TCCA, and structural details are shown in Figure 1b,c. During training, FD-TCL is introduced on top of the cross-entropy loss in the classification branch, as shown in Figure 1a, to provide neurodynamics-inspired temporal regularization for prediction sequences. During inference, the final multiclass motor imagery decisions are obtained after a temporal convolutional network (TCN), a fully connected layer (Dense), an average fusion operation (Average Fuse), and a Softmax layer.

From a functional point of view, the overall framework can be summarized as four logically progressive and mutually cooperating stages, namely feature representation enhancement, spatiotemporal feature coupling, temporal consistency constraint, and channel structure optimization. First, in the feature representation enhancement stage, an LPA module is introduced. In this module, learnable mappings into a latent subspace are constructed for keys and values in the attention mechanism so that alignment and aggregation of attention are carried out in a low-dimensional latent space that is closely related to the task. With this design, discriminative features across combinations of time, frequency band, and channel are emphasized without the need for an additional complex scoring network, numerical stability of attention computation is improved, and computational complexity is reduced.

Second, in the spatiotemporal feature coupling stage, a TCCA module is integrated into the framework. This module adopts a cascade strategy that processes time first and channels afterward and explicitly represents the bidirectional interaction between temporal patterns and channel topology. In the temporal dimension, TCCA captures key temporal patterns through global alignment and dynamic aggregation. In the channel dimension, channels over different brain regions are adaptively reweighted and denoised based on the encoded temporal information. With the aid of this design, the model can represent spatiotemporal structural characteristics of EEG signals in a more refined way and may help reduce the influence of session-related nonstationary variations.

Third, at the level of the training objective, FD-TCL is introduced as an extension of the standard cross-entropy loss. This loss function applies a noncausal and symmetric fractional difference operator to the sequence of class probabilities that evolves along time and imposes smoothness and consistency constraints on the dynamic process of predictions. On the one hand, high-frequency oscillations in prediction sequences are suppressed. On the other hand, the model is encouraged to produce more stable temporal confidence evolution over longer time scales. This design is motivated by the gradual and long-memory characteristics commonly observed in EEG dynamics and should be interpreted as a soft temporal regularization strategy.

Finally, in the channel structure optimization stage, an MPDOA module is employed to perform structured channel selection. This module conducts an evolutionary search that alternates exploration and exploitation in the channel space and identifies compact channel combinations with high information content, while explicit control is imposed on channel redundancy and inference cost under the constraint that validation performance is maintained. At the same time, MPDOA generates a binary channel mask that can be directly connected to the backbone network, compresses the channel dimension at the input side, reduces the overall computational burden, and improves channel-level transparency. The specific mathematical forms and implementation details of these modules are presented in Section 3.1, Section 3.2, Section 3.3 and Section 3.4.

### 3.1. Latent-Projected Attention

In EEG signal modeling, temporal characteristics and cross-channel dependencies constitute one of the most critical types of structural information. Conventional attention mechanisms compute correlations among query (Q), key (K), and value (V) and dynamically assign weights to capture temporal dependencies within a certain range [31]. However, for multichannel and high-dimensional MI-EEG signals, such mechanisms are often insufficient when long-range dependencies and complex cross-channel interactions are involved. The attention distribution tends to concentrate in local neighborhoods, and stable discriminative spatiotemporal patterns over a global range are difficult to extract. Therefore, it is necessary to introduce a more compact representation with structural priors while the adaptive nature of attention is maintained, so that the modeling capacity for complex temporal relationships and inter-channel interactions is enhanced.

For this purpose, an LPA mechanism is constructed inside DO-PI-EATCNet [32]. The core idea is to explicitly introduce a set of learnable latent subspace bases on top of the standard attention structure and to use them as projection bases for K and V. In this way, attention computation is mapped from the original feature space into a lower-dimensional latent subspace that is more consistent with task requirements. During this process, the geometric semantics of attention are preserved, discriminative power is strengthened, and numerical properties of the computation are improved. The standard scaled dot-product attention can be expressed as

(1) $$\mathrm{Attention}(Q,K,V)=\mathrm{softmax}\left(\frac{QK^{T}}{\sqrt{d_{k}}}\right)V,$$

where Q denotes the query, K denotes the key, V denotes the value, and $d_{k}$ denotes the dimensionality of K. This form of attention builds similarity measures entirely on explicit input features and lacks explicit modeling of latent structure. On this basis, the LPA mechanism introduces a learnable latent subspace matrix Z, with $Z\in {\mathbb{R}}^{d_{k}\times d_{z}}$ where $d_{z}$ denotes the dimensionality of the latent subspace. By applying Z to K and V by linear projections, projected representations in the latent subspace are obtained as follows

(2) $$K^{\prime }=KZ,\;V^{\prime }=VZ.$$

Attention is then computed in the latent subspace to obtain the output representations as follows

(3) $$\mathrm{LatentAttn}(Q,K,V,Z)=\mathrm{softmax}\left(\frac{Q{\left(K^{\prime }\right)}^{T}}{\sqrt{d_{k}}}\right)V^{\prime }.$$

As a result, alignment and aggregation of attention are no longer carried out directly in the original representation space of K and V. Instead, they are mapped into a low-dimensional latent subspace that is spanned by Z. The latent basis matrix Z is learned automatically through backpropagation during training and can be regarded as a set of global basis functions that are closely related to the task. These basis functions are used to distill key information across different time steps and channels, so that attention in this subspace may produce more discriminative task-related representations. Compared with explicit additional scoring networks, this design only introduces low-rank linear transformations. Therefore, both the number of parameters and the computational cost remain within a controllable range, and numerical instability of attention scores is alleviated.

In temporal modeling of MI-EEG signals, LPA further enhances the capacity of the model to capture long-range dependencies and cross-channel interactions. On the one hand, basis vectors in the latent subspace can aggregate related information from multiple time steps and multiple channels. In this way, compact representations are formed that capture slowly evolving dynamic patterns and cooperative activity among different brain regions. On the other hand, through low rank reconstruction of K and V, LPA reduces the dimensionality of attention computation while maintaining focus on discriminative information, which decreases the risk of overfitting and numerical oscillation in high-dimensional spaces. In practical applications, LPA maintains high sensitivity to both transient changes and sustained variations. When patterns that are highly related to the task appear in some channels within a certain time interval, the corresponding directions in the latent subspace are strengthened and subsequently influence the attention distribution. As a result, the model may better maintain attention to such task-related patterns over longer temporal ranges, which can contribute to improved multiclass MI-EEG classification performance.

### 3.2. Temporal Channel Cascaded Collaborative Attention

EEG signals are typical temporal data and exhibit pronounced temporal dependencies as well as complex cross-channel correlation structures. Conventional attention mechanisms in practical modeling often focus on only one aspect in either the temporal dimension or the channel dimension, and it is difficult for these mechanisms to fully represent temporal relationships and inter-channel interactions within a unified framework. This limitation becomes more evident in multichannel and high-dimensional MI-EEG scenarios [33]. Therefore, a TCCA is introduced in DO-PI-EATCNet in order to explicitly couple global dependencies along both temporal and channel dimensions within a single network structure and to enhance the modeling capacity for complex spatiotemporal patterns.

TCCA is constructed based on LPA and adopts a two-stage strategy that processes time first and channels afterward. This temporal-first and channel-second order is adopted according to the temporal nature of MI-related EEG rhythms. In motor imagery EEG, discriminative information is mainly reflected by time-varying event-related desynchronization/synchronization (ERD/ERS) dynamics within specific frequency bands. Therefore, it is beneficial to first identify task-related temporal segments and then use the encoded temporal context to refine the spatial contribution of different channels.

This design follows the common processing logic of representative EEG decoding models. For example, EEGNet first applies temporal convolution to learn frequency-specific filters and then uses depthwise convolution to learn spatial filters over EEG channels [34]. Similarly, ShallowConvNet and DeepConvNet adopt temporal convolution followed by spatial filtering, which is analogous to the band-pass filtering and CSP-based spatial filtering pipeline in traditional EEG analysis [35]. MI-EEGNet also follows this temporal-before-spatial design for motor imagery EEG feature extraction [36]. In addition, Temporal and Channel Attention Convolutional Network (TCACNet) emphasizes that both task-related temporal slices and channel-wise information distribution are important for MI-EEG classification [33].

In contrast, a channel-first design performs spatial reweighting before stable temporal evidence is extracted, which may amplify noisy or session-specific channel fluctuations, especially under cross-session EEG distribution shifts. A parallel temporal channel design can model temporal and channel dependencies simultaneously, but the channel branch is not explicitly conditioned on temporally encoded MI dynamics. Therefore, the proposed temporal-to-channel cascade enables channel reweighting to be guided by discriminative temporal representations and provides a clearer physiological motivation for the adopted attention order.

In terms of network architecture, the design follows the idea of a Transformer decoder block and consists of two attention sublayers and one position-wise feedforward network. Layer normalization, residual connections, and dropout are introduced into each sublayer so that stability during training and controllability of representation capacity are ensured. Let the feature tensor that is obtained from the convolutional front-end after appropriate reshaping be denoted as follows

(4) $$X\in {\mathbb{R}}^{C\times T\times d},$$

where C denotes the number of channels, T denotes the number of time steps, and d denotes the feature dimensionality. TCCA takes X as input, applies LPA along the temporal dimension and the channel dimension in sequence, and combines residual connections and layer normalization at each stage so that stable deep representations are formed. The first stage is a temporal attention sublayer, which represents global dependencies over time within each channel. Specifically, layer normalization is first applied along the last dimension of the input features, and the following normalized tensor is obtained as follows

(5) $${\tilde{X}}^{t}=\mathrm{LN}(X),$$

LPA is then applied along the temporal dimension. The query, key, and value of the temporal sublayer are denoted as follows

(6) $$Q_{t}={\tilde{X}}^{\left(t\right)}W_{Q}^{\left(t\right)},\;K_{t}={\tilde{X}}^{\left(t\right)}W_{K}^{\left(t\right)},V_{t}={\tilde{X}}^{\left(t\right)}W_{V}^{\left(t\right)},$$

where $W_{Q}^{\left(t\right)},W_{K}^{\left(t\right)},W_{V}^{\left(t\right)}$ are learnable mappings. In each attention head, a latent subspace basis matrix $Z_{t}$ is used to project the keys and values and representations in the temporal latent subspace are obtained as follows

(7) $$K_{t}^{\prime }=K_{t}Z_{t},\;V_{t}^{\prime }=V_{t}Z_{t}.$$

Attention is then computed in the temporal latent subspace and can be expressed as follows

(8) $${\mathrm{LPA}}_{t}(Q_{t},K_{t},V_{t},Z_{t})=\mathrm{softmax}(\frac{Q_{t}{\left(K_{t}^{\prime }\right)}^{T}}{\sqrt{d_{k}}})V_{t}^{\prime },$$

where $d_{k}$ denotes the dimensionality of the key vector in a single head. The multi-head outputs are concatenated and linearly transformed, and a residual connection with the input is applied to obtain the output of the temporal sublayer as follows

(9) $$X^{\left(t\right)}=X+\mathrm{Dropout}(LPA_{t}(Q_{t},K_{t},V_{t},Z_{t}).$$

This process performs global modeling of relationships among time steps for each channel and emphasizes temporal segments that are more critical for the classification task. At the same time, compression and reconstruction in the latent subspace reduce the dimensionality of attention computation and alleviate numerical instability. The second stage is a channel sublayer, which represents cooperative relationships among channels over different brain regions at each time step. The output of the temporal sublayer, denoted by $X^{t}$, is used as the input, and layer normalization is again applied along the last dimension to obtain the normalized representation as follows

(10) $${\tilde{X}}^{c}=\mathrm{LN}(X^{\left(t\right)}),$$

and the channel dimension is rearranged so that each time step corresponds to a set of channel features, and channel-wise query, key, and value are then constructed along the channel dimension as follows

(11) $$Q_{c}={\tilde{X}}^{\left(c\right)}W_{Q}^{\left(c\right)},\;K_{c}={\tilde{X}}^{\left(c\right)}W_{K}^{\left(c\right)},V_{c}={\tilde{X}}^{\left(c\right)}W_{V}^{\left(c\right)}.$$

Another latent subspace basis matrix $Z_{c}$ is then used to obtain the channel-wise latent representations as follows

(12) $$K_{c}^{\prime }=K_{c}Z_{c},\;V_{c}^{\prime }=V_{c}Z_{c}.$$

The corresponding channel-wise attention is computed as follows

(13) $${\mathrm{LPA}}_{c}(Q_{c},K_{c},V_{c},Z_{c})=\mathrm{softmax}\left(\frac{Q_{c}{\left(K_{c}^{\prime }\right)}^{T}}{\sqrt{d_{k}}}\right)V_{c}^{\prime }.$$

The multi-head channel attention outputs are linearly transformed and passed through a dropout operation, then added to the input through a residual connection to obtain the channel sublayer output as follows

(14) $$X^{\left(c\right)}=X^{\left(t\right)}+\mathrm{Dropout}({\mathrm{LPA}}_{c}(Q_{c},K_{c},V_{c},Z_{c}).$$

Thus, based on temporal patterns already encoded by the temporal sublayer, the channel sublayer adaptively reweights contributions of different channels, realizes dynamic reconstruction and denoising of channel topology, and represents cooperative activation patterns among task-related brain regions with higher accuracy. After the two attention sublayers, TCCA further includes a position-wise feedforward network that enhances nonlinear representation capacity. This feedforward sublayer adopts a pre-layer normalization structure and is given as follows

(15) $${\tilde{X}}^{\left(f\right)}=\mathrm{LN}(X^{\left(c\right)}),H^{\left(f\right)}=\mathrm{FFN}({\tilde{X}}^{\left(f\right)}),$$

where feedforward network (FFN) is a position-wise two-layer fully connected module whose hidden dimensionality is a fixed multiple of the input dimensionality and which is equipped with a nonlinear activation and dropout. The final output of the feedforward sublayer is obtained by a residual connection with the input as follows

(16) $$X^{\mathrm{TCCA}}=X^{\left(c\right)}+\mathrm{Dropout}(H^{\left(f\right)}).$$

With this design, TCCA uses LPA as the basic operator and represents global dependencies along the temporal dimension and the channel dimension in sequence, while layer normalization, residual connections, and a position-wise feedforward network are combined to form a stable structure, which is similar to a Transformer decoder block. This structure facilitates convergence during training and keeps the representation capacity under control. In the processing flow, TCCA first achieves global alignment and dynamic aggregation along the temporal dimension and then performs adaptive reweighting and denoising along the channel dimension. By jointly modeling temporal information and channel information within a single module, TCCA produces more compact feature representations and provides effective inputs for subsequent temporal convolution and classification layers. In this study, the physiological plausibility analysis is centered on selected-channel distributions and scalp topographies, while TCCA is evaluated mainly through its contribution to representation learning and classification performance.

### 3.3. Fractional-Order Difference Temporal Consistency Loss

To improve the temporal stability of segment-level EEG classification, FD-TCL is introduced as a fractional-order temporal consistency regularizer on top of the standard cross-entropy loss. It acts on temporal probability or logit sequences to suppress high-frequency prediction fluctuations. The fractional-order design is motivated by the long-memory and nonstationary characteristics of EEG dynamics, and is positioned as a neurodynamics-inspired regularization strategy [37].

Consider a sequence $x_{1:T}$ of length T, which can be a time-varying representation obtained from multichannel EEG by an encoder. At each time step, the discriminative model outputs a logit vector $l_{t}\in {\mathbb{R}}^{L}$ over L classes, or only a probability vector $p_{t}\in \Delta^{L-1}$ if no logits are directly available, in which case a logit transformation is applied as described later. A segment-level aggregation operator Agg(⋅) produces a segment-level logit $\bar{l}=\mathrm{Agr}(l_{1:T})$, and the segment-level prediction probability is then given by $\hat{p}=\mathrm{softmax}(l)$. Let the ground truth label be y∈{1,…,L}. On this basis, a loss L is constructed that contains a classification term and a fractional-order temporal regularization term. The key idea is to impose a linear convolution-type constraint on temporal sequences by means of a Grünwald-Letnikov (GL) fractional difference operator and to use a symmetric noncausal form to avoid phase shift [38]. GL fractional difference coefficients are first defined. For any order α∈ℝ and any nonnegative integer k, the coefficient is defined as follows

(17) $$a_{k}={\left(-1\right)}^{k}\left(\frac{\alpha }{k}\right),\;\left(\frac{\alpha }{k}\right)=\frac{\alpha (\alpha -1)\cdots (\alpha -k+1)}{k!},\;a_{0}=1.$$

For finite-length sequences, a truncated approximation is used. The maximum truncation order is denoted by M≪T. To avoid causal bias and to reduce phase distortion, a symmetric noncausal fractional difference operator is adopted as follows

(18) $$\Delta_{nc}^{\alpha }s_{t}=\frac{1}{2}\sum_{k=1}^{L_{t}}a_{k}\left(s_{t-k}-s_{t+k}\right),\;L_{t}=\min\left\{M,t-1,T-t\right\},$$

where $s_{t}$ denotes a scalar temporal quantity to which the fractional-order regularization is applied. If the model outputs logits $l_{t}$ is defined as follows

(19) $$s_{t}=l_{t}^{\left(y\right)}.$$

That is, the logit sequence of the true class y is used. If the model only outputs the probability $p_{t}^{\left(y\right)}\in (0,1)$ of this class, a temperature-scaled logit transform is applied as follows

(20) $$s_{t}=\frac{1}{\tau }\log\frac{p_{t}^{\left(y\right)}+\varepsilon }{1-p_{t}^{\left(y\right)}+\varepsilon },$$

where τ>0 is a temperature parameter and ε>0 is a numerical stability term. The symmetric operator reduces to the central difference $\frac{1}{2}(s_{t+1}-s_{t-1})$ when α=1, and $\Delta_{nc}^{\alpha }s_{t}\to 0$ tends to zero when α→0. Therefore, for  0<α<1 it represents long memory smoothing and sparsity with a weak derivative-type behavior. To compensate for energy scaling differences that are caused by boundary truncation, a position-dependent normalization factor is introduced as follows

(21) $$c_{t}=\sqrt{2\sum_{k=1}^{L_{t}}a_{k}^{2}},r_{t}=\frac{\Delta_{nc}^{\alpha }s_{t}}{c_{t}},$$

where $r_{t}$ is regarded as a fractional-order residual. When t is close to the boundaries, $L_{t}$ becomes small, and the introduction of $c_{t}$ makes $\mathrm{Var}(r_{t})$ approximately consistent across different t. The set of valid time indices is denoted by $J=\{t:L_{t}\geq 1\}=\{M+1,\ldots ,T-M\}$ when an effective convolution implementation is used. When mirror padding is adopted to compute residuals over the full sequence, J can be set to {1,…,T}. These residuals are then used to define the fractional-order temporal regularization term, and a quadratic energy penalty is adopted as follows

(22) $$L_{\mathrm{fd}}=\frac{1}{\left|J\right|}\sum_{t\in J}r_{t}^{2}.$$

This term encourages $s_{t}$ to become smoother in a nonlocal central difference sense under GL weights and suppresses high-frequency oscillations in a fractional-order sense. In view of the interpretability requirements in EEG analysis, the alignment term introduces an EEG-derived teacher signal to guide the learned temporal representation toward temporally structured EEG fluctuations. In this study, the teacher signal $\phi {\left(x\right)}_{t}$ is defined deterministically as the *μ*-band log-power envelope extracted from the MPDOA-selected EEG channels. This design avoids introducing an additional teacher network and keeps the temporal reference signal reproducible.

Specifically, for each trial, the EEG signals from the MPDOA-selected channels are first band-pass filtered in the *μ*-band (8–13 Hz). The analytic signal is then obtained using the Hilbert transform, and the channel-wise log-power envelope is calculated as $\log(|H(B_{\mu }(x_{c}(t))){|}^{2}+\epsilon )$, where $B_{\mu }(\cdot )$ denotes *μ*-band filtering, H(⋅) denotes the Hilbert transform, and ϵ is a small constant for numerical stability. The log-power envelope of each selected channel is normalized within each trial, and the normalized envelopes are averaged across the selected channel set to obtain a one-dimensional temporal teacher signal $\phi {\left(x\right)}_{t}$. The obtained $\phi {\left(x\right)}_{t}$ is linearly interpolated to the same temporal length as the student temporal feature. No class labels, test information, or additional teacher network are used to generate $\phi {\left(x\right)}_{t}$. Based on this deterministic teacher signal, the normalized fractional-order residual is constructed as follows

(23) $$u_{t}=\frac{\Delta_{\mathrm{nc}}^{\alpha }\phi {\left(x\right)}_{t}}{\sqrt{2\sum_{k=1}^{L_{t}}a_{k}^{2}}}.$$

The student temporal feature is projected into a one-dimensional temporal sequence by a linear projection layer and normalized using trial-wise z-score normalization. Its normalized fractional-order residual is denoted as $r_{t}$. The alignment loss then constrains the student residual $r_{t}$ to follow the EEG-derived teacher residual $u_{t}$. A scaling coefficient η>0 is introduced to control the alignment ratio between the two residual sequences, leading to the following consistency regularizer

(24) $$L_{\mathrm{align}}=\frac{1}{\left|J\right|}\sum_{t\in J}{\left(r_{t}-\eta u_{t}\right)}^{2}.$$

By combining the above terms with the segment-level cross-entropy, the customized total loss proposed in this section, including an optional weight decay term, is defined as follows

(25) $$L=\underset{L_{\mathrm{CE}}}{\underset{︸}{-\log{\hat{p}}^{\left(y\right)}}}+\underset{L_{\mathrm{fd}}}{\underset{︸}{\lambda \frac{1}{\left|J\right|}\sum_{t\in J}r_{t}^{2}}}+\underset{L_{\mathrm{align}}}{\underset{︸}{\mu \frac{1}{\left|J\right|}\sum_{t\in J}{\left(r_{t}-\eta u_{t}\right)}^{2}}}+\gamma \|\theta {\|}_{2}^{2},$$

where λ,μ, and γ≥0 are hyperparameters and θ is the set of learnable parameters. This formulation couples the segment-level discriminative ability of $L_{\mathrm{CE}}$ with the temporal regularization of $L_{\mathrm{fd}}$ and $L_{\mathrm{align}}$. $L_{\mathrm{fd}}$ suppresses high-frequency fluctuations in prediction sequences and encourages smoother temporal evolution of prediction confidence. When needed, $L_{\mathrm{align}}$ encourages the prediction dynamics to be guided by the EEG-derived *μ*-band temporal reference and provides an EEG-related temporal reference and supports temporal stability during training.

### 3.4. Multi-Population Dream Optimization Algorithm for Channel Selection

In EEG classification tasks, high dimensionality, low signal-to-noise ratio, and nonstationarity often lead to the introduction of redundant channels and irrelevant information. These redundant channels may increase computational cost and weaken classification performance. Therefore, channel selection plays an important role in improving the efficiency and robustness of MI-EEG models. However, conventional channel selection methods usually rely on fixed strategies or heuristic rules. It is still difficult to effectively reduce the number of channels while maintaining classification accuracy. Therefore, flexible and automatic selection of high-information channels remains a critical problem in MI-EEG classification. To address this issue, the idea of dream optimization algorithms [39] is adapted, and an MPDOA algorithm is designed for EEG channel selection. MPDOA is an adaptive channel selection method based on swarm intelligence. It alternates between global exploration and local exploitation in the channel space and searches for compact EEG channel subsets under the constraint of maintaining validation performance.

The overall procedure of MPDOA is illustrated in Algorithm 1, and MPDOA consists of four main stages, namely initialization, fitness evaluation, dream updating, and elite preservation with convergence checking. In the initialization stage, an initial population matrix $X^{\left(0\right)}\in {\left[0,1\right]}^{E\times C}$ of size E is generated, where C denotes the number of EEG channels.

To incorporate weak prior neurophysiological knowledge without imposing a hard constraint on channel selection, a two-level initialization strategy was adopted in MPDOA. First, a core channel set K={C3,Cz,C4} was defined according to the most representative sensorimotor electrodes in the BCI Competition IV-2a montage. These channels correspond to the contralateral hand-related sensorimotor areas and the midline region associated with lower-limb motor imagery. Second, a neighboring priority channel set P={FCz,C1,CP1,CP2,Pz} was defined around these core electrodes. These electrodes provide spatial extension around the central and centro-parietal regions and allow the initialization to account for inter-subject variability in MI-related ERD/ERS distributions. During population initialization, channels in K were assigned the highest initial priority value of 1, channels in P were initialized with moderately high random values sampled from U(0.5,1), and the remaining channels were initialized with lower random values. This strategy only guided the initial population toward anatomically plausible sensorimotor-related regions. It did not constrain the subsequent MPDOA search process, and all 22 EEG channels remained searchable during iteration. Therefore, the final selected channel subset was determined by the fitness-driven MPDOA optimization and could form subject-specific channel configurations. The initialization of the channel-encoding vector was therefore defined as

(26) $$X_{ij}^{\left(0\right)}∼\left\{\begin{array}{ll} 1, & j\in K\\ U(0.5,1), & j\in P\\ U(0,0.5), & j\notin K\cup P \end{array}\right..$$

A thresholding function is then applied to binarize the continuous representation and a channel mask matrix is obtained as follows

(27) $$Z_{ij}^{\left(0\right)}=H(X_{ij}^{\left(0\right)}-0.5),$$

where H(⋅) denotes the Heaviside step function and is defined as follows

(28) $$H(x)=\left\{\begin{array}{lll} 1, & x\geq 0, & \\ 0, & x<0. & \end{array}\right.$$

In the fitness evaluation stage, a corresponding channel subset is constructed for each individual $Z_{i}^{\left(t\right)}$ in each generation, and the original input is cropped along the channel dimension to obtain $X_{S_{i}}$. A classification model with ATCNet as the backbone is then trained on this subset. During training, the custom loss with the fractional-order temporal regularization term defined earlier is adopted so that segment-level discriminative accuracy and temporal prediction stability are jointly considered. The fitness of the *i*-th individual is defined as follows

(29) $$F_{i}=(1-Acc_{i})+\omega P_{i}^{pri}+\varphi P_{i}^{cri},$$

where $Acc_{i}$ denotes the classification accuracy on the validation set, $P_{i}^{\mathrm{pr}i}$ represents a penalty for insufficient preservation of priority channels, $P_{i}^{\mathrm{cr}i}$ represents a penalty for missing critical channels, and ω and φ are penalty weight hyperparameters. If the number of channels selected by an individual is below a prescribed lower bound, for example, fewer than two channels, a large penalty value is directly assigned to discard solutions that are not meaningful in practice. The dream updating stage is used to simulate an alternation between subconscious exploration and memory consolidation. Let $T_{\max}$ be the total number of generations and let the exploration ratio of generation t be denoted by $\xi_{t}=1-t/T_{\max}$, which adjusts the balance between global exploration and local exploitation. For the i-th individual, the update of the continuous encoding vector at generation t+1 is expressed as follows

(30) $$X_{i}^{\left(t+1\right)}=\left\{\begin{array}{lll} X_{i}^{\left(t\right)}+r(\mathrm{rand}-0.5)\times 2, & \left(\mathrm{global}\;\mathrm{exploration}\;\mathrm{phase}\right) & \\ X_{i}^{\left(t\right)}+r(x^{*}-X_{i}^{\left(t\right)})+0.1N(0,1), & \left(\mathrm{local}\;\mathrm{exploitation}\;\mathrm{phase}\right) \end{array}\right.$$

where $x^{*}$ denotes the individual with the best fitness in the current generation, r is a random coefficient, rand is a uniform random variable on [0, 1], and N(0,1) denotes a standard normal perturbation. The exploration phase focuses on large random perturbations in the continuous space in order to enlarge the search region. The exploitation phase moves solutions in a directed way toward the neighborhood of the current optimum and superimposes small random perturbations in order to refine local structures. After each update, every component of $X_{i}^{\left(t+1\right)}$ is clipped to the interval [0, 1] and binarization is then performed by Equation (27) to obtain the new channel mask $Z^{\left(t+1\right)}$. In the elite preservation and convergence checking stage, the individual $x^{*}$ with the best fitness in each generation and its fitness value $F^{*}$ are preserved and directly transferred to the next population to prevent the current optimum from being destroyed by random perturbations during updates. At the same time, changes of the best fitness value over several consecutive generations are monitored, and when these changes remain below a predefined threshold, convergence of the algorithm can be determined in advance, and the iterations can be terminated. The final optimal individual provides a sparse channel selection vector, and the corresponding classification model reports evaluation metrics such as accuracy, Cohen’s *κ*, and confusion matrix, which are used to comprehensively evaluate the trade-off between recognition performance and channel compression of the selected channel subset.


**Algorithm 1.** Pseudo-code of MPDOA.  **Input:** Population size E; max iterations $T_{\max}$; EEG $I\in {\mathbb{R}}^{S\times C\times T}$; labels y; masks p,q; penalties α,β; fractional params $\left(\alpha_{\mathrm{frac}},N_{\mathrm{frac}},\lambda_{\mathrm{frac}}\right)$ **Output:** $x_{\mathrm{best}}\in {\left\{0,1\right\}}^{C}$; best fitness $F_{\mathrm{best}}$; best accuracy ${\mathrm{Acc}}_{\mathrm{best}}$; convergence list C 1: Initialize real population $X^{\left(0\right)}\sim U{\left(0,1\right)}^{C}$; bias $x_{ij}\sim U(0.5,1)$ if $p_{j}=1$; clip to ${\left[0,1\right]}^{C}$ 2: $Z^{\left(0\right)}\leftarrow 1\left(X^{\left(0\right)}>0.5\right)$ 3: **for all**
$z\in Z^{\left(0\right)}$ **do** 4:    **if**
$||z|{|}_{0}<2$ **then** 5:      Acc←0,F←10, **continue** 6:    **end if** 7:    Select channels $S=\{j|z_{j}=1\}$; slice $I\to I_{S}$ 8:    Train ATCNet_ on $I_{S}$, y with loss (25); evaluate Acc 9:    Compute fitness F via (29) 10: **end for** 11: $b=\arg\min_{i}F_{i};x_{\mathrm{best}}=X_{b}^{\left(0\right)};F_{\mathrm{best}}=F_{b};{\mathrm{Acc}}_{\mathrm{best}}={\mathrm{Acc}}_{b};C=[F_{\mathrm{best}}]$ 12: **for**
t=1 to $T_{\max}$ **do** 13:     Update $X^{\left(t\right)}$ by (30); clip to ${\left[0,1\right]}^{C}$; elitism: $X_{I}^{\left(t\right)}=x_{\mathrm{best}}$ 14:     Binarize $Z^{\left(t\right)}=1(X^{\left(t\right)}>0.5)$ 15:     Evaluate $Z^{\left(t\right)}$ as above; $b=\arg\min_{i}F_{i}^{\left(t\right)}$ 16:     **if**
$F_{b}^{\left(t\right)}<F_{\mathrm{best}}$ **then** 17:      $x_{\mathrm{best}}=X_{b}^{\left(t\right)};F_{\mathrm{best}}=F_{b}^{\left(t\right)};{\mathrm{Acc}}_{\mathrm{best}}={\mathrm{Acc}}_{b}^{\left(t\right)};$ 18:     **end if** 19:     Append $F_{\mathrm{best}}$ to C 20: **end for** 21: **return** $1(x_{\mathrm{best}}>0.5),F_{\mathrm{best}},{\mathrm{Acc}}_{\mathrm{best}},C$



## 4. Dataset

### 4.1. Dataset Description

In this study, the public BCI Competition IV-2a dataset is used as the evaluation benchmark [40]. The dataset contains nine healthy subjects, A01–A09. Each subject completed two recording sessions on different days, denoted as Session 1 and Session 2. Each session includes 12 runs, and each run includes 48 trials, resulting in 288 trials per session.

The dataset provides four MI tasks, namely left hand, right hand, both feet, and tongue. In the label definition used in this study, Class 0, Class 1, Class 2, and Class 3 correspond to left-hand, right-hand, both-feet, and tongue motor imagery, respectively. The class distribution inside each session is balanced, and each class contains 72 trials. Therefore, 576 trials are available for each subject across the two sessions, and the total number of trials in the dataset is 5184.

**Figure 2:**
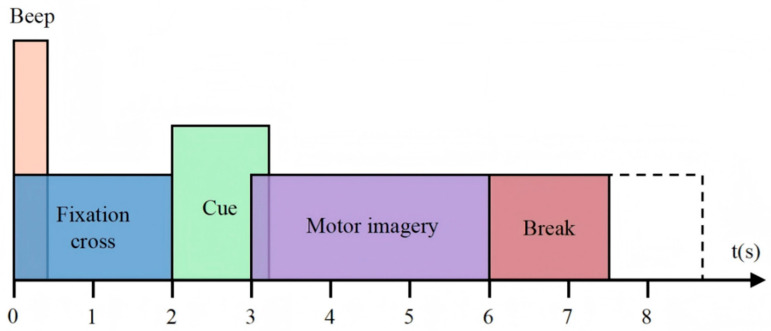
Time sequence of a single trial in the BCI Competition IV-2a motor imagery paradigm.

Signal acquisition was performed with 22 EEG channels and 3 EOG channels using Ag-AgCl electrodes placed according to the international 10–20 system. The sampling rate is 250 Hz. The time course of a single trial is illustrated in Figure 2 and consists of a preparation and fixation period, a cue presentation period, and an MI period of about 4 s.

To evaluate generalization across days, a within-subject cross-session protocol is adopted. Session 1 is used as the training set, and Session 2 is used as the test set. Inside the training set, 10% of the trials are further selected as a validation set by stratified sampling over classes. All experiments keep the labels and trial order provided by the organizers of the competition unchanged, ensuring that the experimental setup and results are reproducible.

### 4.2. Data Preprocessing

To obtain a unified and reproducible input representation, three EOG channels are first removed from the original recordings, and only 22 EEG channels are retained. EEG signals are then re-referenced by using a common average reference (CAR) to reduce common-mode noise and global drifts. A zero-phase filtering pipeline is subsequently constructed, in which a 50 Hz notch filter and a 4–40 Hz bandpass filter are applied in sequence so that MI rhythms in the μ and β related frequency bands are preserved. Filter order and transition bandwidth are determined according to the sampling rate and window design criteria so that the required passband specifications are satisfied while an approximately zero group delay is maintained.

After filtering, all trials are read from the training session A0{subject}T and the test session A0{subject}E. With the cue onset as reference, a time window from 1.5 s to 6.0 s of the original 7 s signal of each trial is extracted and an MI segment of 4.5 s is obtained. Under the sampling rate of 250 Hz, each segment contains 1125 time points and each single-trial sample has the size 22 × 1125. The whole dataset can therefore be represented as number of trials × number of channels × number of time steps. All 22 EEG channels are retained at this stage.

The data are then divided into a training set and a test set according to the experimental protocol. A subject-specific cross-session setting is used as the default configuration and support is reserved for extended schemes such as leave-one-subject-out (LOSO) cross-validation. After data splitting, a standardization transformer StandardScaler is fitted separately for each channel of each subject on the training set to perform zero-mean and unit-variance normalization. The same scaling parameters are then applied to the validation set and the test set of the same subject so that data leakage caused by information from the test set during fitting is avoided.

### 4.3. Evaluation Metrics

The performance of the model is evaluated by three metrics, namely accuracy, Cohen’s *κ*, and confusion matrix [41]. Let the multiclass predictions on the labeled test set be represented by a confusion matrix $H=[h_{ij}]\in Q^{L\times L}$, where $h_{ij}$ denotes the number of samples whose true class is i and predicted class is j. Here, L is the total number of classes and $Q=\sum_{i,j}h_{ij}$ is the total number of test samples. Accuracy is defined as the proportion of samples that are predicted correctly among all samples and can be written as follows

(31) $$\mathrm{Acc}=\frac{1}{Q}\sum_{i=1}^{L}h_{ii}.$$

To further consider the effect of chance agreement, the Cohen’s *κ* is also computed. Let $p_{o}=\mathrm{Acc}$ denote the observed agreement and let $p_{e}$ denote the expected agreement under random prediction, which is defined as follows

(32) $$p_{e}=\frac{1}{Q^{2}}\sum_{i=1}^{L}\left(\sum_{j=1}^{L}h_{ij}\right)\left(\sum_{j=1}^{L}h_{ji}\right).$$

It represents the expected chance agreement under the assumption that predictions and true labels are statistically independent. The Cohen’s *κ* is then defined as follows

(33) $$\kappa =\frac{p_{o}-p_{e}}{1-p_{e}}.$$

when model predictions agree with the true labels only by chance, κ is approximately zero, and κ equals one when the predictions are completely consistent with the labels. This metric remains sensitive to discriminative ability even when the class distribution is imbalanced or the overall accuracy is high.

## 5. Results and Analysis

### 5.1. Model Configuration and Training Settings

To accommodate the coupled temporal, frequency, and spatial characteristics of four-class MI-EEG, the overall model adopts ATCNet as the backbone and consists of three parts, namely a convolutional front-end, an LPA-TCCA module, and a temporal convolutional network TCN. Segment-level decisions are obtained by a sliding window fusion strategy. Structural details of each module and the configuration of the sliding windows are summarized in Supplementary Table S1. The model input is an EEG tensor of size batch size × 1 × 22 × 1125, which corresponds to a 4.5 s segment with 22 channels for each trial. The convolutional front-end performs one-dimensional convolutions and depthwise separable convolutions along the temporal dimension and combines them with pooling operations in order to obtain lightweight spatiotemporal representations. On this basis, LPA-TCCA applies latent subspace multi-head attention along the temporal dimension and the channel dimension and models global dependencies across time and channels. A shallow TCN then performs further temporal modeling, expands the temporal receptive field, and controls computational complexity. In the classification stage, a fixed number of overlapping sliding windows is placed along the temporal axis, and the whole trial is divided into multiple subsegments. Features of each window are successively processed by LPA-TCCA and TCN, and a fully connected layer produces a window-level prediction at the end. Segment-level four-class probabilities are finally obtained by taking the arithmetic means of the outputs over all the windows.

Training and regularization-related hyperparameters are summarized in Supplementary Table S2. The loss function consists of a segment-level cross-entropy term and a temporal consistency regularizer based on GL fractional-order difference, which corresponds to FD-TCL. L2 regularization is applied to convolutional layers and fully connected layers, and norms of convolutional kernels are constrained, which improves generalization ability and numerical stability. The model is trained with the Adam optimizer for at most 1000 epochs under fixed learning rate and batch size settings. The computational platform consists of an Intel Core i9-12900K processor Intel Corporation, Santa Clara, CA, USA), 64 GB of memory, and an NVIDIA GeForce RTX 3080 graphics processor (NVIDIA Corporation, Santa Clara, CA, USA) with compute capability 8.6 and approximately 7.4 GB of available memory. Training and inference are implemented in Python 3.10 by using TensorFlow-GPU 2.10.1.

### 5.2. Experimental Results

#### 5.2.1. Convergence Characteristics and Cross-Session Classification Performance

**Figure 3:**
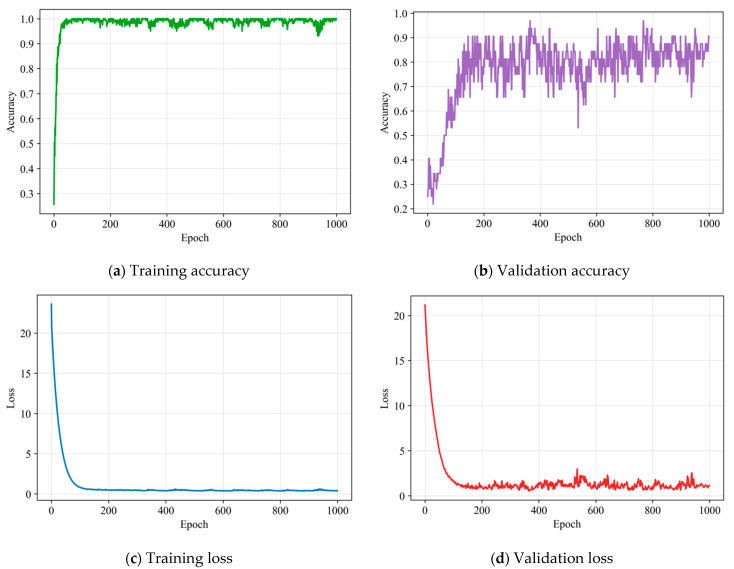
Accuracy and loss curves of training and validation for the model.

To gain deeper insight into the optimization process and generalization properties, Figure 3 presents the curves of loss and accuracy during training and validation. It can be observed that the main learning phase is completed within approximately the first 100 epochs, during which the training loss decreases rapidly from about 22 to below 1 and the training accuracy increases steadily from about 0.25 and approaches 1.0. After this stage, validation performance enters a relatively stable region between about 150 and 200 epochs. Validation accuracy remains roughly in the range from 0.75 to 0.90, and validation loss exhibits small fluctuations around 1.0. Although transient spikes in the loss are observed in the later part of training, around epochs 550 and 850, these perturbations disappear within a few subsequent epochs and do not lead to persistent overfitting or performance degradation. Overall, the training and validation curves suggest a stable optimization process under the tested within-subject cross-session setting, without persistent overfitting or sustained performance degradation.

**Figure 4:**
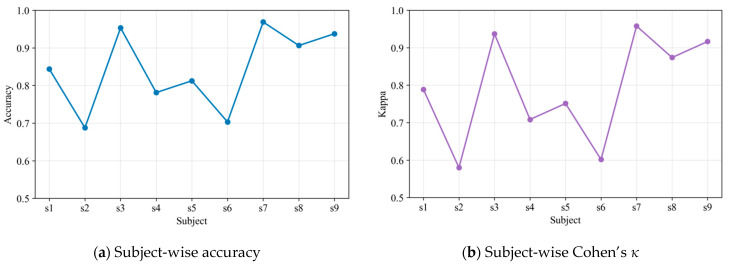
Comparison of classification accuracy and Cohen’s *κ* across subjects.

To evaluate the performance of the proposed method in a cross-session setting, comprehensive experiments were conducted on the BCI Competition IV-2a dataset by using a protocol in which Session 1 was used for training and Session 2 was used for testing. Figure 4 shows four-class MI-EEG results for nine subjects. On the group level, the average accuracy of the model was 0.844 ± 0.106, and the average Cohen’s *κ* was 0.790 ± 0.142, where values are given as mean ± standard deviation. The corresponding 95% confidence intervals were [0.763, 0.925] and [0.682, 0.899]. At the individual level, performance varied to some extent. For four of the nine subjects, accuracy exceeded 0.90. Subjects S7 and S3 were representative cases, with accuracy and Cohen’s *κ* of 0.969 and 0.958 for S7 and 0.953 and 0.937 for S3, which indicates that discriminative features of MI tasks were captured effectively for these subjects. In contrast, subjects S2 and S6 were relatively difficult cases, with accuracy values of 0.688 and 0.703 and Cohen’s *κ* of 0.580 and 0.602. These lower scores may be related to individual physiological differences, noise levels of the signals, or insufficient consistency in task execution. Despite inter-subject variability, group-level performance remained stable. Coefficients of variation of accuracy and Cohen’s *κ* were 12.5 percent and 17.9 percent, which indicates that classification performance for most subjects lay in a similar range. In addition, accuracy and Cohen’s *κ* showed a high correlation, indicating that the subject-wise accuracy trends were consistent with the chance-corrected agreement measure.

**Figure 5:**
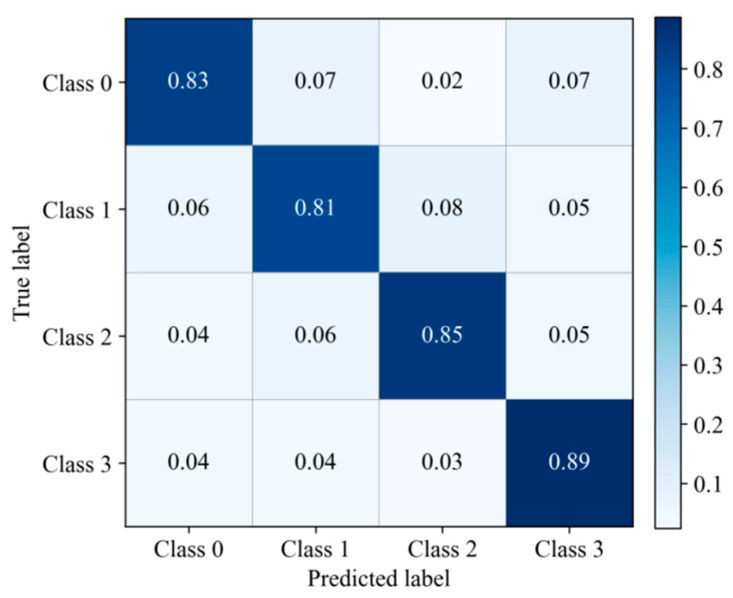
Confusion matrix of the model for four-class MI-EEG classification.

To visualize discriminative performance of the model in the four-class task, the overall confusion matrix is shown in Figure 5. For clarity, Class 0, Class 1, Class 2, and Class 3 denote left-hand, right-hand, both-feet, and tongue motor imagery, respectively. The predictions are mainly concentrated along the diagonal, which indicates that the classes are correctly distinguished in most cases and that interclass misclassification is relatively limited. For Class 0, the prediction accuracy is 0.83, and misclassified samples are mainly assigned to Class 1 with proportion 0.07 and Class 3 with proportion 0.07. For Class 1, the prediction accuracy is 0.81, and misclassifications occur mainly as Class 0 with proportion 0.06 and Class 2 with proportion 0.08. For Class 2, the prediction accuracy is 0.85, and misclassifications are concentrated in Class 1 with proportion 0.06 and Class 3 with proportion 0.05. Class 3 achieves the highest prediction accuracy of 0.89, with relatively few misclassifications, which occur mainly as Class 0 with a proportion of 0.04 and Class 1 with a proportion of 0.04.

Overall, the confusion matrix indicates effective discriminative performance under the tested within-subject cross-session multiclass MI-EEG setting. To more directly examine the remaining confusion between neighboring or closely related MI classes, a pairwise confusion analysis was further conducted based on the normalized confusion matrix. For a pair of classes i and j, the symmetric pairwise confusion rate was calculated as $PCR(i,j)=\frac{1}{2}(M_{ij}+M_{ji})$, where $M_{ij}$ denotes the proportion of samples from class i misclassified as class j. Based on this calculation, the highest pairwise confusion was observed between Class 1 and Class 2, with a symmetric confusion rate of 7.0%, followed by Class 0 and Class 1 with 6.5%, and Class 0 and Class 3 with 5.5%. The confusion between Class 2 and Class 3 was also noticeable, indicating that residual errors were concentrated in several MI class pairs with relatively similar or partially overlapping sensorimotor representations. These results suggest that finer discrimination among neighboring or closely related body-part imagery classes remains challenging, although the overall diagonal dominance of the confusion matrix is preserved.

#### 5.2.2. Comparison with Existing Methods

To assess the overall advantage of the proposed model under cross-session conditions, several representative convolutional and temporal modeling frameworks were selected as comparison methods. All experiments in Table 1 were conducted under a unified protocol with identical data preprocessing, data partitions, train-test splitting, validation strategy, evaluation metrics, and stopping criteria. For clarity, all baseline results in Table 1 were obtained by reimplementing and retraining the compared methods under the same experimental protocol used in this study.

Table 1 reports the reproduced classification results on the BCI Competition IV-2a dataset under the within-subject cross-session setting. Overall, the proposed model achieves an average accuracy of 84.4% and an average Cohen’s *κ* of 0.790 over the nine subjects. It obtains the best combined performance among all compared methods, with clear improvements over traditional convolutional networks and attention-enhanced models.

From the viewpoint of method categories, classical shallow networks such as ShallowConvNet [43] and EEGNet [34] can reach high accuracy for some individual subjects, but their overall accuracy and stability are less satisfactory. Models based on temporal convolutions, namely EEG-TCNet [44] and TCNet Fusion [45], provide improved temporal modeling, although a noticeable performance bottleneck remains under the cross-session setting. ATCNet [46] is a representative lightweight baseline model, and its average accuracy and kappa under the same protocol are 81.1% and 0.748. In comparison, the proposed model improves mean accuracy by about 4.1% relatively and improves kappa by about 4.9% relatively.

From the subject-level perspective, the proposed model maintains relatively high accuracy for all nine subjects. For S7 to S9, accuracy exceeds 90%, which indicates relatively stable performance across subjects under the tested within-subject cross-session protocol. For the relatively difficult subjects S2 and S6, the proposed model still outperforms the baseline methods, suggesting improved performance for relatively difficult subjects under the tested within-subject cross-session protocol. The overall standard deviation of accuracy is 9.96, which is lower than that of most competing approaches.

In addition to the methods evaluated under the unified cross-session protocol in Table 1, recent high-performing MI-EEG models were also reviewed to provide broader state-of-the-art context. TCFormer [48] and SATrans-Net [49] report accuracies of 84.79% and 84.72% on BCI IV-2a, respectively, showing competitive performance of recent Transformer-based EEG decoders. SST-DPN [20] and MBMANet [50] report 84.11% and 83.18%, respectively, representing competitive prototype-learning and attention-enhanced CNN architectures. Because these recent studies used different preprocessing pipelines, temporal windows, augmentation strategies, or evaluation protocols, their reported results are presented only as reference comparisons and are not included in Table 1. Compared with these recent architectures, the proposed model remains competitive in classification accuracy and provides additional advantages in channel reduction and channel-level anatomical plausibility.

#### 5.2.3. Ablation Studies and Computational Efficiency

Because the proposed framework contains multiple components, the ablation study was designed to clarify their distinct functional roles and to examine how each module contributes to different aspects of the model. LPA, TCCA, and FD-TCL are mainly performance-oriented components, whereas MPDOA is mainly an efficiency- and compactness-oriented channel selection component. Therefore, the effect of MPDOA should be evaluated jointly in terms of classification accuracy, selected channel number, and computational load, instead of relying only on accuracy. To avoid ambiguity in the ablation settings, each variant was constructed by modifying only the specified module while keeping the remaining training protocol unchanged. The ATCNet baseline used the original ATCNet backbone with all 22 EEG channels and the standard cross-entropy loss. The “ATCNet + MPDOA” variant used the MPDOA-selected subject-specific channel subset as the input to the original ATCNet backbone, while LPA, TCCA, and FD-TCL were removed. This variant was added to isolate the pure effect of channel selection without advanced attention modules or the proposed loss function.

The “ATCNet + LPA” variant inserted a standalone Latent-Projected Attention block into the ATCNet backbone to evaluate the effect of latent-space attention enhancement alone. The “ATCNet + TCCA” variant inserted the Temporal Channel Cascaded Collaborative Attention block into the backbone. Since TCCA uses LPA as its internal attention operator, the “ATCNet + TCCA” variant includes only the LPA operations required inside the temporal and channel attention sublayers of TCCA, but does not include an additional standalone LPA enhancement block. Therefore, “ATCNet + LPA” evaluates standalone latent attention enhancement, whereas “ATCNet + TCCA” evaluates the temporal-first and channel-second cascaded attention structure. The “ATCNet + LPA + TCCA” variant contains both the standalone LPA enhancement block and the TCCA block. The “ATCNet + FD-TCL” variant retained the original ATCNet structure but replaced the standard objective with FD-TCL. The remaining combined variants were constructed by progressively adding LPA, TCCA, FD-TCL, and MPDOA.

As shown in Table 2, the ATCNet baseline achieves an average accuracy of 81.1% and a kappa of 0.748. When MPDOA is applied alone to the original ATCNet backbone, the accuracy decreases to 79.3% and kappa decreases to 0.718. This result indicates that direct channel compression may remove part of the useful information when the baseline representation capacity is limited. Therefore, MPDOA alone does not necessarily improve classification accuracy, but it provides a compact input structure and isolates the effect of channel selection.

After LPA is added as a standalone enhancement block, the accuracy increases to 82.3%, indicating that latent-space attention improves discriminative feature representation. When TCCA is added alone, the accuracy further increases to 83.0%. Since this variant uses LPA only as the internal attention operator of TCCA and does not include an additional standalone LPA block, the observed improvement mainly reflects the benefit of the temporal channel cascaded structure. FD-TCL also improves the baseline performance, reaching 83.5% accuracy and a kappa of 0.780, suggesting that the fractional-order temporal consistency regularization is beneficial for within-subject cross-session prediction stability.

When LPA and TCCA are combined, the accuracy increases to 84.2%, showing that standalone latent attention enhancement and temporal channel cascaded modeling are complementary. Adding FD-TCL further improves the accuracy to 84.9% and kappa to 0.798. Finally, after MPDOA is incorporated into the full model, the accuracy slightly decreases from 84.9% to 84.4%, while kappa remains high at 0.790. This small decrease is acceptable because MPDOA substantially reduces the number of input EEG channels and improves the compactness and interpretability of the model. These results indicate that the final model achieves a good trade-off among classification accuracy, channel reduction, and channel-level compactness.

After the contribution of each component is clarified, a computational resource comparison is further provided to evaluate the efficiency aspect of the proposed framework. Since DO-PI-EATCNet is developed based on the ATCNet backbone and further incorporates LPA, TCCA, FD-TCL, and MPDOA for cross-session discriminability, temporal stability, and channel compactness, the comparison is not intended to demonstrate absolute lightweight superiority over all EEG decoding models. Instead, it provides a more complete assessment of the trade-off among classification performance, channel compactness, parameter size, arithmetic complexity, and inference latency. Table 3 presents a comparison between the proposed model before and after MPDOA and the seven reimplemented baseline models reported in Table 1 in terms of parameter size, arithmetic complexity, inference latency, and channel reduction.

All measurements were obtained under the same hardware and test conditions, using an RTX 3080 GPU (NVIDIA Corporation, Santa Clara, CA, USA), a batch size of 1, 5 warm-up runs, and 30 repeated inference runs. Table 3 compares the proposed model before and after MPDOA with the seven reimplemented baseline models reported in Table 1 in terms of parameter size, arithmetic computational complexity, and runtime latency.

As shown in Table 3, lightweight convolutional models such as EEGNet, EEG-TCNet, FBCNet, ShallowConvNet, and TCNet-Fusion require fewer parameters and lower inference latency than the proposed model. This is because these models are mainly based on compact convolutional and temporal convolutional structures. However, as reported in Table 1, their average cross-session classification performance is lower than that of the proposed method under the same experimental protocol. In contrast, EEG Conformer has the largest parameter size and computational complexity among the compared methods, with 871.8 K parameters and 74.10 M MACs. Compared with EEG Conformer, the proposed model requires substantially fewer parameters and MACs while achieving better average classification performance.

For the proposed framework, MPDOA mainly affects input-side channel compactness and arithmetic complexity. As shown in Table 3, introducing MPDOA reduces the number of EEG channels from 22 to 15 and decreases MACs from 31.05 M to 22.74 M, corresponding to an approximately 26.8% reduction. The parameter size remains nearly unchanged, decreasing only from 175.6 K to 175.3 K, because MPDOA performs channel selection rather than internal network pruning. The mean inference latency changes slightly from 5.57 ms to 5.72 ms, suggesting that the reduced MACs do not directly translate into latency acceleration under the current batch-size-one GPU setting, possibly due to the sliding-window strategy and attention-related execution overhead.

Together with the ablation results in Table 2, these findings further support the role of MPDOA as an efficiency-oriented channel-compression module. Compared with the uncompressed LPA-TCCA-FD-TCL variant, the MPDOA-based model maintains comparable classification performance, with accuracy changing from 84.9% to 84.4%, while reducing the number of input channels and MACs. This indicates that MPDOA improves channel compactness and arithmetic efficiency with only a limited impact on classification performance.

#### 5.2.4. Sensitivity Analysis of FD-TCL Hyperparameters

To evaluate the sensitivity of FD-TCL to its key hyperparameters, a one-factor-at-a-time sensitivity analysis was conducted. Specifically, α, η, and μ were varied separately, while the remaining two parameters were fixed at their default values. The default setting was α=0.6, η=0.5, and μ=0.3. The tested values were α∈{0.4,0.6,0.8}, η∈{0.25,0.5,0.75}, and μ∈{0.1,0.3,0.5}. The same training and evaluation protocol as the main experiment was used. As shown in Table 4, the default setting α=0.6, η=0.5, and μ=0.3 achieves the highest accuracy among the tested configurations. When α is changed to 0.4 or 0.8, the accuracy decreases to 79.5% and 81.2%, respectively, indicating that the fractional order affects the temporal regularization strength and scale of FD-TCL. However, the performance does not collapse under nearby values, suggesting that the method is not dependent on a single extremely narrow setting. Similar trends are observed for η and μ, where moderate values provide better performance than overly small or large values. These results indicate that FD-TCL remains reasonably stable within the tested parameter ranges, while the default configuration provides the best overall performance.

#### 5.2.5. Channel Selection Behavior and Cross-Session Performance

**Figure 6:**
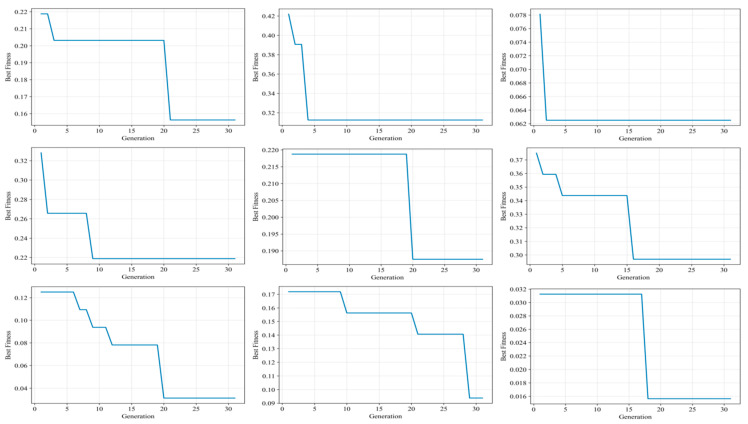
Fitness convergence curve of the MPDOA algorithm.

To further analyze the behavior of MPDOA during optimization and its stability across subjects, convergence trajectories of channel search and spatial distribution and classification performance of the finally selected channels are examined separately. Through this analysis, it is possible to verify whether channel selection actually reduces redundant input while preserving or even enhancing cortical information that is related to MI tasks. The convergence properties of MPDOA are first investigated at the algorithm level. Figure 6 shows the variation of fitness with the number of generations during channel selection. In the first 5–10 generations, the best fitness decreases rapidly, which indicates that the algorithm can effectively discard clearly redundant channel combinations in the early stage and quickly approach a better channel subset. Subsequently, the rate of decrease in fitness gradually slows, and the curve enters a stable region, where an overall monotonically convergent trend is observed, and only small local oscillations appear. This behavior indicates that MPDOA can complete a transition from coarse search to fine adjustment within a limited number of generations and provides high search efficiency while maintaining good stability and convergence in the later stage.

**Figure 7:**
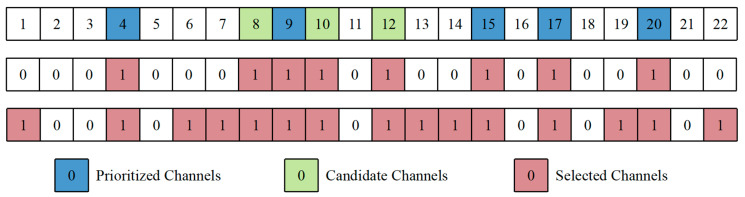
Illustration of prioritized, candidate, and selected EEG channels by MPDOA.

After convergence characteristics have been verified, channel selection results are further examined from the perspectives of spatial distribution and cross-session behavior. Figure 7 schematically illustrates three types of channels in the search space of MPDOA. Blue squares indicate prioritized channels, green squares indicate candidate channels, and red squares indicate selected channels that are automatically determined by MPDOA. This visualization helps to provide an intuitive understanding of how the algorithm balances preservation of task-critical brain regions and compression of redundant inputs. Channels near prioritized regions tend to be retained and expanded, whereas channels that are far from task-related areas are more likely to be discarded during the search.

To evaluate the effectiveness of MPDOA in cross-session EEG classification, channel selection results and spatial distribution consistency across subjects are further examined. Table 5 summarizes channel selection results for nine subjects on the BCI Competition IV-2a dataset, including cross-session four-class accuracy, kappa, the number of selected channels, and indices of selected channels. Overall, optimal channel subsets of different subjects are spatially concentrated around C3, C4, Cz, CPz, and neighboring sensorimotor areas, which is consistent with the classical somatotopic pattern of MI tasks. For some subjects, such as S3, S7, and S9, a clear set of preferred channels is formed in these regions. Cross-session accuracy for these subjects exceeds 90% and Cohen’s *κ* remains at a high level, which indicates that MPDOA preserves task-critical brain regions while effectively discarding redundant channels with limited discriminative contribution. For relatively difficult subjects, the number of selected channels increases slightly, although the subsets still center on the sensorimotor cortex and its neighboring areas. This spatial consistency suggests that the selected channels show channel-level anatomical plausibility and that MPDOA tends to retain sensorimotor-related electrodes while reducing redundant input channels and preserving most of the classification performance.

To further examine the influence of MPDOA search-scale settings, an illustrative sensitivity analysis was conducted on subject S5 using the ATCNet + MPDOA configuration. The preprocessing pipeline, backbone network, training strategy, and evaluation metrics were kept unchanged, while the MPDOA population size, iteration number, and random seed were varied within the tested range. The tested settings included population size/iteration combinations of 5/15, 10/30, and 15/45. Each repeated run was initialized with four different random seeds to examine the effect of stochastic initialization. Across repeated experiments, the mean accuracy was 78.59%, with a standard deviation of 0.35 percentage points. This small variation suggests that the classification performance of MPDOA remained stable under the tested search-scale settings.

The stability of the selected channel subsets was further evaluated using the mean pairwise Jaccard similarity. For any two selected channel sets $S_{i}$ and $S_{j}$, the Jaccard similarity was calculated as $J(S_{i},S_{j})=|S_{i}\cap S_{j}|/|S_{i}\cup S_{j}|$, where $\left|S_{i}\cap S_{j}\right|$ is the number of commonly selected channels between two runs and $\left|S_{i}\cup S_{j}\right|$ is the number of channels selected in either run. For multiple repeated experiments, the Jaccard similarities were calculated over all pairwise combinations and then averaged. The mean pairwise Jaccard similarity was 0.824, indicating a high overlap among the selected channel subsets across repeated MPDOA runs. This result suggests that MPDOA produced relatively stable channel-selection results under different random initializations and search-scale settings. Although this S5-based analysis does not exhaustively quantify all possible MPDOA configurations, it provides preliminary evidence that MPDOA is not highly sensitive to the tested population size, iteration number, and random seed settings.

#### 5.2.6. Channel-Level Anatomical Plausibility and Feature Visualization

**Figure 8:**
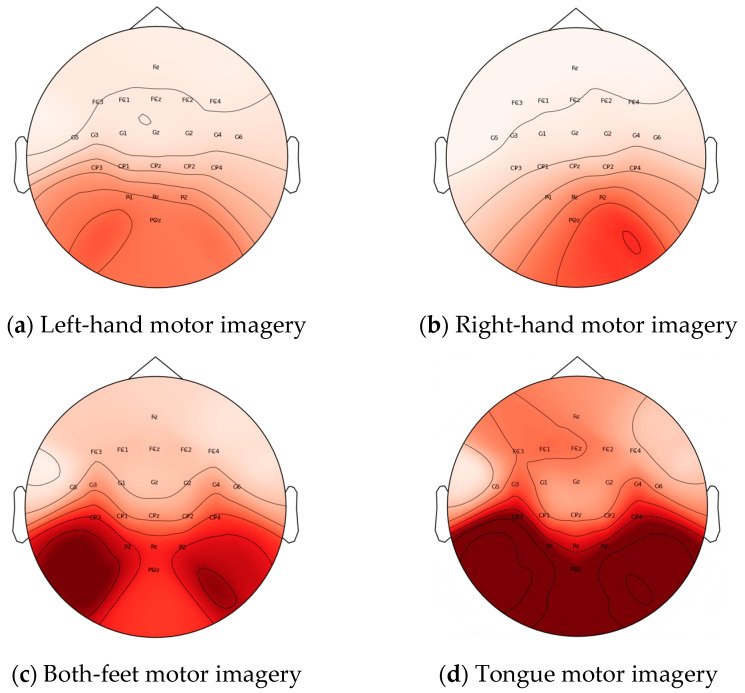
Topographic distributions of μ-band power for four motor imagery tasks.

It should be noted that the interpretability analysis in this study mainly focuses on channel-level anatomical plausibility. Specifically, scalp topographies and selected-channel distributions are used to examine whether the MPDOA-selected electrodes are broadly consistent with expected MI-related sensorimotor regions. These analyses establish a channel-level interpretation basis, while future work may further examine the neurophysiological relevance of the internal attention mechanisms. Building on the previous analyses of cross-session classification performance, confusion matrices, and channel ablation, which have shown clear advantages of the model in numerical metrics, accuracy and kappa alone still cannot answer what the model has learned and whether the learned patterns are consistent with neurophysiological mechanisms. In this section, scalp topography and feature space visualization are used to analyze the μ-band 8–13 Hz and high-dimensional representations. In this way, the behavior of DO-PI-EATCNet and the MPDOA channel selection strategy is examined through channel spatial distribution and feature visualization. Figure 8 presents scalp topographic maps of *μ*-band power for the four MI tasks. Spatial patterns are highly consistent with the classical somatotopic organization of the sensorimotor cortex. For left hand MI in Figure 8a, pronounced event-related desynchronization ERD [51] is observed over the right central and parietal areas around C4 and CP4, which reflects contralateral control of left hand movements by the right hemisphere.

For right hand MI in Figure 8b, a similar ERD pattern appears over the left central and parietal areas around C3 and CP3. The spatial distribution forms an approximately mirrored pattern relative to the left-hand task, and contralateral characteristics are clearly visible. For both feet MI in Figure 8c, activation is mainly concentrated over the midline central and parietal region around Cz and CPz. This pattern shows a typical midline distribution and agrees with the view that movements of both feet are represented in the medial sensorimotor cortex. For tongue MI in Figure 8d, relatively diffuse power changes are observed over bilateral anterior inferior regions such as FC1 and FC2 and extend toward frontal and temporal areas. This pattern is consistent with the fact that cortical representation of tongue movements is relatively widespread and shifted toward anterior regions. Taken together, ERD and ERS patterns show that task related rhythms are distributed over sensorimotor cortex and its neighboring regions. This observation provides a neurophysiologically plausible background for analyzing the selected channels, because the observed *μ*-band spatial patterns are broadly consistent with expected MI-related sensorimotor distributions.

**Figure 9:**
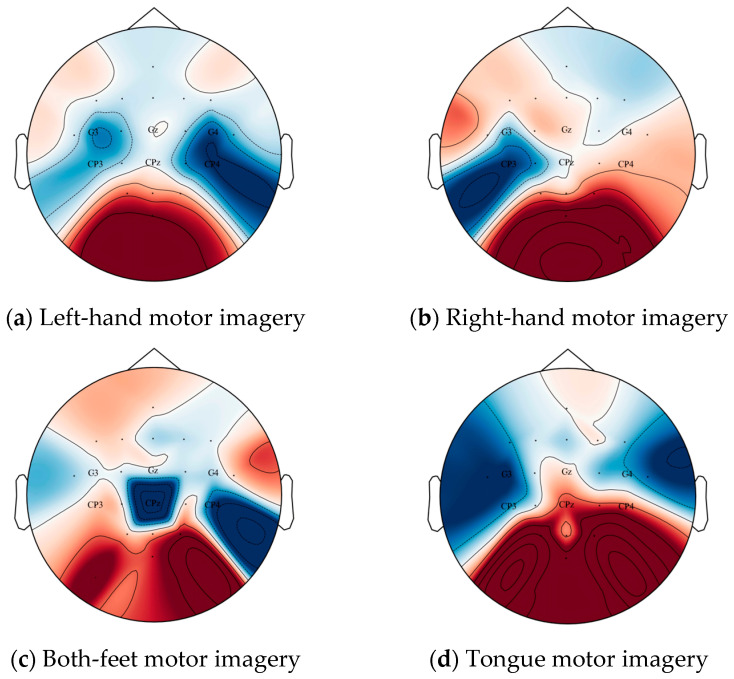
Class-wise μ-band ERD/ERS topographic maps for four motor imagery tasks.

To provide a clearer neurophysiological interpretation of the channel-selection results, class-wise μ-band ERD/ERS topographic maps were generated for the four MI tasks, including left-hand, right-hand, both-feet, and tongue motor imagery. Different from a task-pooled selected-versus-unselected power-difference visualization, the class-wise ERD/ERS maps characterize task-specific *μ*-band modulation relative to the baseline period. ERD/ERS was calculated as $10\log_{10}(P_{task}/P_{base})$, where $P_{base}$ denotes the μ-band power within 0.5–2.0 s and $P_{task}$ denotes the μ-band power within 4.0–6.0 s. This representation allows the selected-channel patterns to be compared with the expected ERD/ERS distributions of each specific MI task. As shown in Figure 9, the class-wise *μ*-band ERD/ERS maps exhibit task-related spatial characteristics over sensorimotor areas. For left-hand motor imagery, stronger ERD appears over the right central and centro-parietal region around C4/CP4, which is broadly consistent with contralateral sensorimotor involvement. For right-hand motor imagery, a clearer ERD pattern is observed over the left sensorimotor region around C3/CP3. For both-feet motor imagery, the ERD/ERS distribution shows central and centro-parietal involvement around Cz/CPz, together with broader bilateral sensorimotor modulation. For tongue motor imagery, the spatial pattern is more diffuse and bilateral-central, which is consistent with the less lateralized representation of tongue-related motor imagery. Overall, these class-wise ERD/ERS topographies provide qualitative support for the channel-level anatomical plausibility of the MPDOA-selected channels.

**Figure 10:**
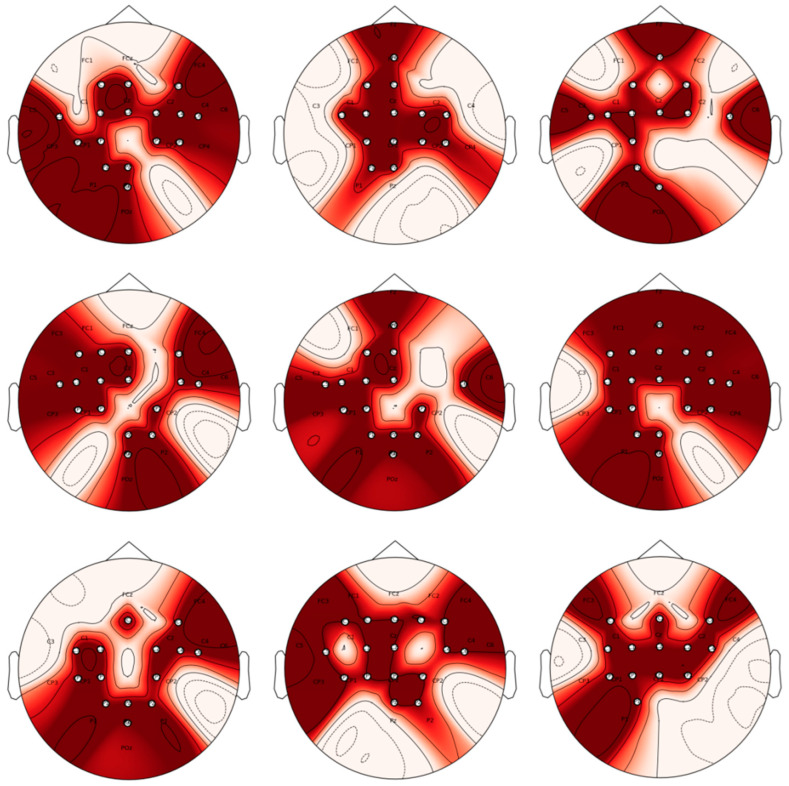
Topographic distribution of selected EEG channels by MPDOA across nine subjects.

Based on the power difference analysis, Figure 10 further characterizes spatial patterns of the binary masks produced by MPDOA by using topographic maps of selected channels for nine subjects. For most subjects, highlighted electrodes are concentrated over C3 and C4, CP3 and CP4, Cz and CPz and their neighboring regions, which closely overlap with somatotopic areas of the sensorimotor cortex. In contrast, relatively fewer selected channels are observed over frontal or parieto-occipital regions that are only weakly related to MI. For some subjects, a clear mirror-like structure appears in contralateral hand regions, and midline and anterior temporal regions that are related to both feet and tongue tasks are also covered to an appropriate extent. This pattern indicates that the selected channels broadly cover sensorimotor regions related to the four MI tasks. The observed spatial consistency shows that MPDOA can stably give priority to core channels over the sensorimotor cortex across subjects and can compress the input dimensionality at the source while classification performance is maintained and the most informative brain regions are emphasized.

**Figure 11:**
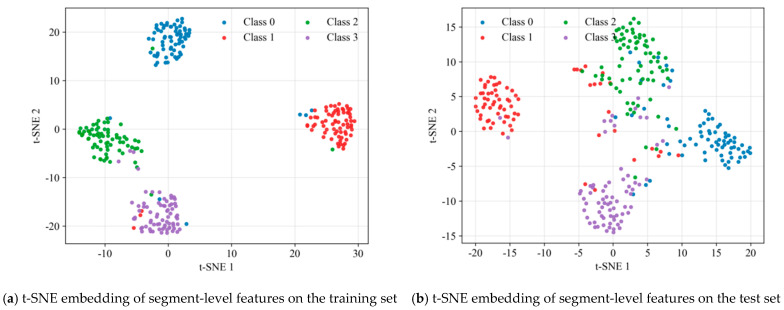
t−SNE visualization of segment-level features on the training and test sets.

However, scalp spatial distributions alone do not describe the feature distribution learned by the model. Therefore, t-SNE is used as a qualitative visualization tool for segment-level representations from the penultimate layer of DO-PI-EATCNet [52]. t-SNE is a nonlinear dimensionality-reduction method that projects high-dimensional representations into a low-dimensional space while preserving local neighborhood relationships. In this study, it is used as a descriptive tool for illustrating the distribution of learned representations. Figure 11a corresponds to training-set features, and Figure 11b corresponds to test-set features. Each point represents one trial in the low-dimensional embedding space, and different colors indicate different MI classes. In the training-set visualization shown in Figure 11a, samples from the same class tend to form class-related groups in the two-dimensional embedding space. The four MI classes show a degree of separation, although several samples still appear near neighboring class regions. This visualization suggests that the learned training representations contain class-related structure. This pattern is consistent with the diagonal dominance observed in the confusion matrix and provides qualitative support for the class-related structure of the learned representations.

In the test-set visualization shown in Figure 11b, class-related grouping is still observable, but the groups become less compact and show local overlaps. These overlaps are mainly located near adjacent boundaries between Class 1 and Class 2 and between Class 2 and Class 3, which is consistent with the misclassification patterns among neighboring body-part imagery classes in the confusion matrix. Overall, the t-SNE visualization provides a qualitative indication that part of the class-related structure is preserved in the unseen session, although local overlaps still exist under cross-session distribution shifts. By combining scalp topographies, channel mask distributions, and t-SNE visualizations, the results provide qualitative support for the numerical findings. The scalp and channel-selection analyses suggest channel-level anatomical plausibility of MPDOA, whereas the t-SNE visualization provides a descriptive illustration of feature distributions learned by DO-PI-EATCNet. Therefore, the visualization results provide complementary qualitative support for understanding the learned feature distributions and the channel-level anatomical plausibility of the selected electrodes.

Previous EEG studies have suggested that establishing a direct link between model attention and neurophysiological states requires dedicated experimental measurements and validation [53]. Since this study does not directly analyze the neurophysiological correlates of the internal LPA/TCCA attention weights, the interpretability claim is limited to channel-level anatomical plausibility and qualitative feature visualization.

## 6. Conclusions

A lightweight framework named DO-PI-EATCNet is proposed for within-subject cross-session MI-EEG decoding, aiming to balance classification performance, channel compactness, and computational efficiency. The model uses ATCNet as the backbone and introduces LPA, TCCA, FD-TCL, and MPDOA with distinct functional roles. LPA and TCCA mainly improve spatiotemporal discriminative representation, FD-TCL serves as a neurodynamics-inspired temporal regularizer, and MPDOA searches for compact subject-specific EEG channel subsets. Under the within-subject cross-session protocol on the BCI Competition IV-2a four-class MI dataset, the final compact DO-PI-EATCNet achieves an average accuracy of 84.4% and a Cohen’s *κ* of 0.790. The performance improvement over representative baselines is mainly attributed to LPA, TCCA, and FD-TCL, as indicated by the ablation results. Compared with the uncompressed LPA-TCCA-FD-TCL variant, the MPDOA-based compact model slightly decreases accuracy from 84.9% to 84.4% but reduces the number of input EEG channels from 22 to about 15 and decreases MACs by approximately 27%. Therefore, MPDOA should be interpreted as an efficiency-oriented channel-compression component. The physiological plausibility discussed in this work mainly refers to the channel-level anatomical consistency between MPDOA-selected electrodes and expected MI-related sensorimotor regions, while the physiological interpretation of the internal attention mechanisms remains a direction for future work.

It is worth noting that the current validation is limited to a within-subject cross-session protocol on the BCI Competition IV-2a dataset. Therefore, the reported results should be interpreted as evidence of cross-day generalization within the same subject, and further validation is needed to assess cross-subject transfer and real-world deployment robustness. Some confusion remains between MI classes of neighboring body parts. The added S5-based sensitivity analysis showed that MPDOA maintained stable performance under the tested search-scale settings, achieving a mean accuracy of 78.59 ± 0.35% and a mean pairwise Jaccard similarity of 0.824 for selected channel subsets. This provides preliminary evidence that MPDOA has reasonable reproducibility within the tested range. Future work will further examine its search-scale effects across more subjects and broader parameter configurations.

Future work may combine individual-specific cortical topology with graph neural or graph attention architectures, evaluate the model on additional datasets and cross-subject transfer settings, explore online adaptive training and learnable fractional orders under low-label or even label-free conditions, and further compress the number of channels and the model size under constraints of wearable acquisition and edge computing.

## Figures and Tables

**Table 1:** Classification performance of different methods in cross-session experiments on the BCI Competition IV-2a dataset.

Methods	S1	S2	S3	S4	S5	S6	S7	S8	S9	Avg.	Std.
FBCNet [42]	86.5	61.5	91.7	75.7	69.1	58.0	88.2	82.6	86.1	77.7	11.60
ShallowConvNet [43]	**88.9**	62.9	95.1	79.2	74.3	68.8	91.3	88.5	89.6	82.1	10.60
EEGNet [34]	88.5	66.0	95.1	73.6	75.4	64.2	90.3	85.8	86.5	80.6	10.47
EEG-TCNet [44]	84.0	66.3	94.1	72.6	76.0	65.9	89.9	84.7	85.4	79.9	9.55
TCNet Fusion [45]	86.1	66.0	93.4	72.6	79.9	66.7	90.3	85.8	85.4	80.7	**9.48**
ATCNet [46]	86.1	64.9	92.1	80.6	71.5	68.8	95.1	83.0	87.9	81.1	9.99
EEG Conformer [47]	**89.9**	58.7	92.7	**80.9**	72.2	65.6	94.4	87.5	88.2	81.1	12.03
Proposed model	84.4	**68.8**	**95.3**	78.1	**81.3**	**70.3**	**96.9**	**90.6**	**93.8**	**84.4**	9.96

**Table 2:** Ablation study results of different module configurations on the classification performance of the model.

Methods	Accuracy (%)	Cohen’s κ
ATCNet (w/o all)	81.1	0.748
w/MPDOA	79.3	0.718
w/LPA	82.3	0.761
w/TCCA	83.0	0.773
w/FD-TCL	83.5	0.780
w/LPA + TCCA	84.2	0.789
w/LPA + TCCA + FD-TCL	84.9	0.798
w/LPA + TCCA + FD-TCL + MPDOA	84.4	0.790

**Table 3:** Computational resource comparison of the proposed model and representative baseline methods.

Model Variant	Channels	Params (K)	MACs (M)	Latency Mean (ms)	GPU Mem Δ (MB)
ATCNet (w/o MPDOA)	22	175.6	31.05	5.57	$1.7\times 10^{-3}$
ATCNet (w/MPDOA)	15	175.3	22.74	5.72	$1.7\times 10^{-3}$
EEGNet	22	2.6	13.14	0.43	$1.1\times 10^{-5}$
EEGTCNet	22	4.3	6.85	1.2	$1.9\times 10^{-5}$
ShallowConvNet	22	38.2	13.27	0.93	$4.9\times 10^{-4}$
TCNet_Fusion	22	17.6	20.71	0.80	$2.3\times 10^{-4}$
EEG Conformer	22	871.8	74.1	3.2	$5.6\times 10^{-3}$
FBCNet	22	11.8	7.13	1.1	$6.5\times 10^{-5}$

**Table 4:** Effects of different FD-TCL hyperparameter settings on cross-session classification accuracy.

Parameter	α	η	μ	Accuracy (%)
Default	0.6	0.50	0.3	84.4
α sensitivity	0.4	0.50	0.3	79.5
α sensitivity	0.8	0.50	0.3	81.2
η sensitivity	0.6	0.25	0.3	82.6
η sensitivity	0.6	0.75	0.3	80.9
μ sensitivity	0.6	0.50	0.1	80.4
μ sensitivity	0.6	0.50	0.5	81.7

**Table 5:** Classification performance and selected EEG channels for each subject.

Subject	1	2	3	4	5	6	7	8	9
Accuracy	84.4	68.8	95.3	78.1	81.3	70.3	96.9	90.6	93.8
Cohen’s *κ*	0.788	0.580	0.937	0.708	0.750	0.603	0.958	0.873	0.916
Selected number	16	14	15	16	15	19	14	16	13
Selected channels	(3, 4, 6, 7, 9, 10, 11, 12, 13, 14, 15, 17, 18, 19, 20, 22)	(1, 3, 4, 8, 9, 10, 11, 12, 15, 16, 17, 18, 19, 20)	(1, 4, 6, 7, 8, 9, 10, 12, 13, 14, 15, 17, 19, 20, 22)	(2, 3, 4, 6, 7,8, 9, 10, 12, 13, 14, 15, 17, 20, 21, 22)	(1, 3, 4, 7, 8, 9, 10, 13, 14, 15, 17, 19, 20, 21, 22)	(1, 2, 3, 4, 5, 6, 8, 9, 10, 11, 12, 13, 14, 15, 17, 18, 19, 20, 22)	(4, 6, 8, 9, 11, 12, 13, 14, 15, 17, 19, 20, 21, 22)	(2, 3, 4, 5, 6, 7, 9, 10, 12, 13, 14, 15, 16, 17, 20, 21)	(2, 4, 6, 8, 9, 10, 11, 12, 14, 15, 16, 17, 19)

## Data Availability

The data presented in this study are available on request from the corresponding author.

## Supplementary Materials

The following supporting information can be downloaded at: https://www.mdpi.com/article/10.3390/s26113336/s1, Table S1: Network Structure and Sliding Window Configuration. Table S2: Training and Loss Function Hyperparameters.
