# Supplementary material for: DO-PI-EATCNet: Efficient-Attention- and Dream-Optimization-Based Channel Selection for EEG Motor Imagery Classification
Source: Sensors (Basel). 2026 May 24;26(11):3336. doi: 10.3390/s26113336 (PMC13258918; doi:10.3390/s26113336)
Supplement: Supplementary file 1 [file sensors-26-03336-s001.zip › sensors-4280929-supplementary.pdf]

Supplementary Table S1. Network Structure and Sliding Window Configuration

Module	Parameter	Value
Input Configuration	Number of Channels	22
	Sampling Rate	250 Hz
	Single Trial Duration	4.5 s (1.5-6.0 s)
	Time Steps	1125
Convolution Front-End	Base Number of Channels	16
	Temporal Convolution Kernel Length	64
	Depthwise Convolution Multiplier	2
	Output Feature Dimension after Convolution Front-End	32
	Pooling Method and Stride	Average Pooling, Stride 7
	Dropout Rate	0.3
LPA-TCCA Module	Number of Temporal Latent Variables	16
	Attention Configuration	mhlatent-cascade-latent
	Number of Transformer-style LPA-TCCA Blocks per Sliding Window	1
	Number of Cascaded Latent-Attention Sublayers	2
	Number of Channel Latent Variables	16
	Number of Heads	2
	Single Head Key Vector Dimension	8
	Total Attention Projection Dimension	16
	Input Feature Dimension of LPA-	32

	TCCA Block	
	Feedforward Expansion Factor	2
	Feedforward Hidden Dimension	64
	Output Feature Dimension of LPA-TCCA Block	32
	Attention and Feedforward Dropout Rate	0.3
Temporal Convolutional Network	Network Depth	2
	Convolution Kernel Length	4
	Number of Channels	32
	Output Feature Dimension before Dense Layer	32
	Activation Function	ELU
	Dropout Rate	0.3
Discrimination and Sliding Window	Number of Sliding Windows	5
	Segment-Level Fusion Method	Probability Averaging

Supplementary Table S2. Training and Loss Function Hyperparameters

Module	Parameter	Value
Regularization and Constraints	Fully Connected Layer L2 Coefficient	0.5
	Convolution Layer L2 Coefficient	0.009
	Convolution Kernel Norm Upper Bound	0.6
Optimization and Training	Optimizer	Adam
	Learning Rate	1×10^{-3}
	ε	1×10^{-7}

	Batch Size	32
	Maximum Training Epochs	1000
Fractional Order Loss	Fractional Order α	0.6
	Truncation Order N	16
	Regularization Weight λ_{fd}	0.2
	Alignment Ratio Coefficient η	0.5
	Alignment Weight μ	0.3
	Temperature Parameter	1.0
	Stability Term	1×10^{-6}
